# Considerations on the Nature and Treatment of Some Hereditary or Family Diseases

**Published:** 1809-04

**Authors:** M. Portal

**Affiliations:** Professor of Medicine in the College of France, and of human Anatomy in the Museum of Natural History, Member of the National Institute, &c. &c.


*Considerations on the Nature and Treatment of some hereditary or Family Diseases; by* M. PORTAL, *Professor of Medicine in the College of France, and of human Anatomy in the Museum of Natural History, Member of the National Institute, &c. &c.*

( Concluded from our last. )

AS to the diseases of Dentition, of which so many infants die in one and the same family, if the cause be investigated, we shall find it frequently in rachitis, and more or less indicated by the vicious conformation of the bones of the cranium in general, and of those of the face in particular. I have found on opening these subjects, remarkable indurations in the brain; and frequently, when other parts of this viscera were soft, that its volume was considerably increased, the circumvolutions being entirely effaced, the ventricles full of water, and a considerable effusion between the membranes of the brain in general.

Similar appearances are daily discovered in the lungs of those who die of scrophulous pulmonary phthisis, a disease which I have showed to be hereditary in another work.

The individuals of a family affected in this manner are destined to die of pulmonary consumption by an hereditary predisposition of the organs. *Quasi jure parentum,* says the great Fernelius, *tabida stirpe sati.* And this disposition, as I have often seen upon dissection, is known swelling of the lymphatic glands of the body in general, and of the lungs in particular, by means of the gelatine and albumen which frequently concrete there, as well as in the cellular texture of the lungs around these glands, and in other places; whence steatomatous concretions result, which end in a bad suppuration, with a more or less extensive destruction of the substance of the lungs.

Several consumptive patients have died before this destruction took place, and without having expectorated any thing, which is not surprising. Others have never expectorated, although there were various steatomatous ulcers in the lungs, but without doubt from the want of a communication between these points of suppuration and the bronchiæ.

Some families are visited with hereditary mesenteric, hepatic, and splenic phthisis, and these affections are the effects of a steatomatous tendency, as may be proved upon dissection.

All these hereditary phthises, although affecting various organs, proceed from the same cause.

In some cases an external eruption fortunately saves the patient from death, which would have been otherwise inevitable.

We have seen diseases of the brain, and particularly of the chest, cured by abscesses formed in the parotid glands, or in the arm-pits; we have also seen diseases of the abdomen completely cured by eruptions in the lower extremities. If we read the works of Pringle, Lieutaud, and other celebrated physicians and surgeons, to whom we may add all our own countrymen, we shall find convincing proofs of these assertions.

Frequently also, hereditary diseases are supplanted by others. We have seen in one and the same family, one child maniacal and the other epileptic, while both died of apoplexy.

These changes or permutations of the brain appear less astonishing, when we reflect that anatomists have generally recognised similar alterations of this organ in subjects that have died of apoplexy or epilepsy, of mania, or stupor. We cannot allow ourselves to think, however, that one and the same cause produces effects so various; we ought rather to conclude, that on some occasions we cannot ascertain the differences.

But the hereditary diseases of the brain are sometimes succeeded by others, having their seat more or less removed from this organ, or rather they succeed to the latter if they do not exist already.

Many diseased persons have died of hydrops pectoris, and other dropsies, who would have died of pulmonary consumption if they had lived longer, their lungs having been found full of steatomatous concretions. Persons have died of internal hemorrhage, which has been well ascertained, when the ordinary symptoms of pulmonary consumption were also present.

In families, the individuals of which have died of pulmonary diseases, there are epileptic patients with malconformations of the cranium. I know a small town in the department of Paris, where some families are succesively visited with mania, epilepsy, and pulmonary consumption; sometimes, however, these diseases are supplanted by others. In a family at Paris, several of the ancestors in which had died of phthisis pulmonalis, two children died of of the same disease, who were under my care. A third who had all the appearances of perishing in the same way has survived, but is very much hunchbacked, and has not since evinced any symptoms of diseased lungs.

I could mention other families attacked by pulmonary consumption, some of the individuals of which became deformed in the back, but escaped from the disease with which they were originally threatened. On the other hand, I know another family, the individuals in which, to the number of seven, are hunch-backed, and alive, and in which two children died of scrophulous pulmonary consumption.

These examples deserve to be recorded, although they do not warrant the idea that all these deformities of shape can secure against the effects of pulmonary consumption, for we find, on the contrary, that they arise sometimes before and at other times after the disease of the lungs has commenced its ravages. In the cases mentioned, however, and others of this description, the scrophulous tendency has its limits, and its effects may be weakened or destroyed by subsequent treatment.

These kinds of steatomatous diseases are therefore propagated in families under the same, or under various forms; and this being the case, may we not suppose that this is the cause, (if not the sole one at least the most common and best known,) of the various configurations in families, and of hereditary diseases, as is proved by the result of the observations we have made?

In proof of this opinion we shall add, that of the diseases which are propagated in families, and *under their true form*, the scrophulous is of all others the best known. *Eo autem terribilius est hoc malum quod a parentibus ad parentes sæpe transit,* said the celebrated Mead, *et hæreditate quam cæpit haud facile se privari sinit.**

When we use the expression, *their true form*, we include among swellings, suppurations and ulcerations of an inveterate nature in the glands of the neck, and other external lymphatic glands, such as those of the arm-pits, the groin, &c. But a scrophulous habit may exist without these external marks; it frequently exists in the mesentery, without affecting the glands of the neck; and it was also in the mesentery that the ancients fixed the immediate seat of it. *Notabis*, says Riolanus, following the

 opinions

* Mead Monita, De Strumis.

opinions of various authors who preceded him, *mesenterium, strumarum radicem ac fundamentum esse, nec foras erumpere unquam, nisi mesenterium strumosum fuerit.**

But this assertion is too general, the mesentery not being always swelled with steatomatous concretions in patients which have similar swellings in other parts of the body. To this Riolanus has confined himself in his *Manuel Anatomique*, where he contents himself with saying, that it is very rare to see scrophulous eruptions to any extent without the mesentery being also scrophulous.

It is now ascertained, that there is no part of the human body which may not be affected with scrophula. We may read the excellent observations of Morgagni† on this subject, and those related by other authors. We may also find some interesting papers on the subject among the Memoires de l'Academie de Chirurgie.

It is, perhaps, one of the most fatal errors in practice, to refuse to admit of the existence of scrophulous, venereal, and scorbutic diseases, except when they affect the parts where we are accustomed to see them break out; observations, infinite in number, having proved that these same parts had not been affected in patients who evidently died from the ravages of one or other of these diseases upon the essential organs of life. In short, when I consider that we find in those who die of the diseases I have mentioned, the same appearances as in persons attacked by the evil, and in various organs, I cannot refrain from regarding the scrophulous vice as the principal and best ascertained cause of these hereditary diseases, without however pretending to deny the existence of others, but are not indicated by any sign, at least that we are yet acquainted with.

But may we not say, that asthma, dropsy, gout, and stone, which are common to some families, and which medical men have, for this reason, comprehended among hereditary diseases, proceed from the same cause, or at least in some measure partake of it? This does not appear equally evident at first sight, because their transmission in families is not so frequent, nor have they such immediate relation with the scrophulous tendency.

It is not rare, however, to observe in these diseases, that

* Anthropogr. Lib. II. in fol. edit. Paris, 1649, p. 108. — Mes Observations sur le rachitisme, p. 185.

† Epist. Art. 27, 28, 29.

that the gelatine and albumen are more or less affected with thickening, or any other alteration, as in various hereditary diseases. Who knows but that asthma is generally occasioned by various concretions of the lungs, and principally by swellings of the lymphatic glands in general, and of the bronchiæ in particular, this disease being frequently at the same time united with a defect of configuration in the bony system.

We often discover in those who die of dropsy, gelatinous and albuminous swellings and indurations in the brain, the lungs, the liver, and other organs, but we particularly find them upon the dissection of a person who has died of hereditary dropsy; and it cannot be doubted that there are dropsies of this description. Besides this, the albumen in dropsies in general, and still more particularly in the hereditary dropsy, is concrete, and forms polypous bodies in the cavities of the heart, in the blood vessels, and particularly in the veins. This hereditary dropsy is frequently the effect of a vicious tendency, which concretes the albumen, and deprives it of serosity; a cause similar to that which we have ascertained in the other hereditary diseases, of which we have already treated.

But is it not possible that the gout and stone, which are common in some families, attacking the same individuals at once, or succeeding each other, in different persons of the same family, should proceed from a cause similar to that which produces hereditary diseases?

It is certain, with respect to the gout, that it is frequently united to rachitis, the most common, or at least the best ascertained of all hereditary diseases. The extremities of the bones in gouty persons, forming the articulations, are swelled, and their substance is sometimes soft, and sometimes brittle, like the bones of ricketty persons. We may add that, in general, the bones of gouty persons decrease in weight in proportion as the arthritic congestions are considerable, as if they were deprived of the substance which should proceed to form the bones in general, and which has been diverted from them in order to seize upon the articulations. Thus there are relations between the gout and rachitis, which it is impossible to mistake.

It remains to be ascertained if there is any relation between the stone and the gout. Both are formed by congeries, the basis of which is as it were formed of a muco-albuminous substance, more or less concrete, with which several other extraneous substances are united. Observation has proved, that the gout and stone frequently attack the same individual; this has been remarked by medical practitioners in all ages; they have also remarked, that asthma, terminating in hydrothorax, often supervenes.

From what has been said, it appears that hereditary diseases are more or less connected with scrophula; and, *in the first place,* rachitis, pulmonary consumption, epilepsy, and other diseases of the brain, particularly when accompanied by malconformation of the cranium; *in the second place,* dropsy, asthma, gout, stone, &c.

But if hereditary or family diseases proceed from nearly similar causes, why are they not all developed at the same period of life? In natural history and in medicine, there is a multitude of well authenticated facts, for which we cannot assign a satisfactory reason, and the latter are rather of this number: it is certain, however, that the dropsy in the brain, or hydrocephalus, is peculiar to children in the state of infancy.

Convulsions are very frequently the effects of laborious dentition.

The formation of sores, or the evil in the neck, generally takes place about the age of 7, or sometimes at the age of puberty, being the periods of life at which the epileptic affections generally display themselves.

Scrophulous pulmonary phthisis carries off the individuals in families attacked by it, from the age of 18 to 33, 34, and sometimes later, for children often die of this disease before their parents, who frequently follow them as victims to the same malady.

Dropsy of the chest, of the belly, or anasarca, being the frequent effects of steatomatous swellings of the lungs and abdominal viscera, prove fatal to individuals from 40 to 60 years of age.

Apoplexy and paralysis produce death about the same age, and sometimes later. Thus hereditary diseases break out at periods, more or less distant from infancy: Although there is nothing constant to be depended on in this respect, the general result is not less worthy of being remarked.

But what is the nature of the scrophulous taint, which occasions so many and various hereditary or family diseases? The difficulties on this subject are multiplied in proportion to our eagerness to discover some doctrinal points from which to set out in the healing art. We are as little acquainted acquainted with the nature of the scrophulous taint as we are with scorbutus, lues venerea, &c.*

We are acquainted with their effects alone; dissections having frequently presented to anatomists the same appearances in those who died of lues venerea, as in those who were truly scrophulous. We know that the venereal disease, both when well and when ill treated, has been followed by scrophulous affections; and it is from reiterated observations of this description, that some medical men, both among the ancients and the moderns, have not hesitated to propose the same remedy for the treatment of scrophula and lues venerea: *Lues venerea et strumæ et elephas, aliquid habent cognatum,* says Baillon, in some parts of his works; and in others, *Affines sunt lues venerea, strumæ et elephas.* Astruc has also established, that the scrophulous taint was frequently a degenerated syphilitic taint; and Bouvart, Baden, Lalouette, and other eminent physicians and surgeons, have latterly furnished new proofs on this point, which have several times directed them to fortunate practical results.

For these Fifty years past, we have had at Paris so remarkable a proof of the degeneration of the venereal virus into steatomatous and rickety diseases, that it is scarcely necessary to bring them to the recollection of our readers.

Every person must have been struck with the great number of children attacked with swellings in the abdominal viscera, with large and deformed heads, curvatures of the spine, distortions of the limbs, contraction of the cavity of the chest, some of whom died of consumption and convulsions, or remained in a state of mental imbecility. In the

 bodies

* Fernel contents himself with saying, with respect to the propagation of elephantiasis: *Tantæ est divinæ illius procreatricis facultatis energia, ut in semine intemperato ac prorsus impuro consistens: corporis partes pingat.* Fernel, Pathol. de Elephant. cap. XIX. first column, edit. Paris, 1579. Baillon says, somewhat reservedly, on this subject, *Semini enim nescio quæ vis impressa est, quæ ut valet ad speciem, ita latenter morborum diathesis devehit et transfundet: eaque vis insita est tanquam tradux, ut macrocephali a macrocephalis generentur; et ita lustrare oportet intestinas partes.* Baillon Opera Omnia, tom. III. p. 267. *Consil. med. lib.* II. *consil.* L. Edmund Meara, in his short treatise *De pathologia hereditaria,* contents himself with attributing the cause of hereditary diseases to a thick heterogenous matter in the blood and prolific liquor: an explanation certainly without a meaning; but this practitioner thought, that by destroying this cause, the transmission of disease from father to son would be prevented. Montaigne, who allowed of hereditary diseases, thinking he had the gout of his father, amused himself with witticisms upon the awkward explanations given by medical men of the transmission of diseases from parents to children. All this only proves, that we can seldom give a good explanation of a well authenticated fact.

bodies of some of these children, swellings have been ob served in the lymphatic glands of the lower part of the face, of the neck, arm-pits and groin; and, finally, in some of them we have found pustules on the skin, with chancres on the lips and parts of generation. As most of these children were nursed in the country, it was never doubted that they had contracted these diseases from their nurses. It was discovered, that a great number of these children had been nursed at Montmorency, and the places adjacent; the Government thought proper to send two medical gentlemen, in order to discover the cause of the evil, and if possible, to avert its progress. M. Morand, sen. and M. Lassonne, Members of the Academy of Sciences, were charged with this mission. They discovered in the nurses, traces of the venereal disease, more or less degenerated; they were put upon a strict regimen, and they became healthy and capable of furnishing better milk. The evil was thus checked in its source. Most of the children were treated with mercurials joined to antiscorbutics, and those with whom the disease was not too long rooted, were cured; even their limbs re-assumed the proper shape: but those who were not well cured, and who afterwards married, probably gave birth to children who were diseased like themselves, or perhaps worse. This seems to be beyond a doubt, and it is also very probable, that the nature of their diseases will be more difficult of discovery, if the venereal taint be not manifested in the parts of generation, but by various other symptoms.

What happens, with respect to hereditary rachitis, phthisis, mania, epilepsy, &c. is exemplified to us daily in persons who consult us, and who know well that their parents were affected with the same diseases of which they complain, but the cause of which they are totally ignorant.

What country is there, to which this observation is not applicable? Are there not nations, among whom the human species degenerate by similar causes, more or less ascertained? It is generally understood, that this is the case throughout Spain; several eminent practitioners of that country have assured me, that no where are greater numbers of rickety, consumptive, epileptic, and even maniacal patients to be seen.* We could mention places in

* M. d'Aranda, the Ambassador from Spain, has frequently told me, that a medical quarantine would be highly beneficial to a great part of the inhabitants of some Provinces in Spain,

France, where these diseases abound; Paris, Lyons, Orleans, Reziers, and other great cities, are examples of this kind.

A town in the department of Paris, which I have already mentioned, and which is full of these various evils, more or less resembling scrophula, was originally infected by two or three bad marriages. The children of these connections intermarried, and thus the hereditary diseases were successively multiplied. These examples, more and more evince the propriety of watching over marriages, to prevent effects so direful to the human race: *Quam præclarè humano generi consultum videretur,* said Fernel, *si soli parentes bene habiti, atque sani, liberis operam darent.* De Causis Morborum, lib. I. cap. xi. But when precautions of this description have not been taken, as is too frequently the case, we ought to endeavour, by a proper treatment, to avert from the children the evils to which they were devoted at their birth; and various facts, well authenticated by experience, hold out to us prospects of complete success.

Convinced of the advantages of mercurial preparations against hereditary scrophulous diseases, I was not surprized, at the commencement of my own practice, to find them prescribed in Rachitis by the celebrated Bovart. We know the great use which has been made in this disease of the syrup of Bellet, a preparation of this kind. I adopted it in the early part of my practice, in similar cases, with astonishing success; but, under some circumstances, not having succeeded equally well, I found that this was occasioned by the rachitis being more or less complicated with the scorbutic taint, either from the venereal virus having degenerated into it, as is generally the case when of long standing, or from the latter having been essentially united with the venereal taint when it was contracted, or otherwise.

I joined to the anti-venereal remedy, the most approved antiscorbutics; the lowness and bad digestion, added to the debility of the patients, determined me to give the bitters also.

These remedies were prescribed in various proportions, according to circumstances; sometimes trusting to mercurials alone, I prescribed very small doses of antiscorbutics; at other times I left off the mercurials and adhered to the latter, and frequently I used the bitter remedies freely, freely,* with bathing, and sometimes blistering or cautery; always recommending a rich diet and proper exercise.

---

* These remedies have been prescribed under a great variety of forms; sometimes slight mercurial frictions at intervals, or frequently repeated, have been recommended, while the patient should at the same time take, every morning or evening, one or two spoonsful of antiscorbutic syrup, and sometimes bitter syrup, wine, powders, or pills, of the same description, before dinner. At other times, bitter pills or extracts have been prescribed, with some grains of sweet mercury, the antiscorbutic juices, or the syrup or wine immediately upon rising, or at other periods of the day.

The solution of corrosive sublimate in pure water, mixed with some mild or depurative drink, so that the patient may take a tenth or an eighth of a grain daily, and during a longer or shorter time, accordingly as it is advisable to insist on mercurials. The venereal taint being more or less inveterate, the syrup of Cuisiniere has been given in very small doses, as well as that of Bellet and other mercurial syrups, all these remedies containing more or less mercury.

Combined with the use of antiscorbutics and bitters, taken at the same time or at different periods of the day, these remedies have been efficacious, but particularly when they have been varied and prescribed according to the doses indicated by the nature of the disease and the disposition of the patient. Thus, in order to simplify the treatment and avoid mistakes, particularly with infants, we may be permitted to combine mercurials with antiscorbutics and bitters in the mixture, in the form of syrup. The advantages derived from these remedies, however pharmaceutically informal, have not been inferior to those already obtained when they have been prescribed separately. After having administered this remedy to a multitude of infants, and after having printed an octavo volume full of successful cases, (*Observations sur la Nature et la Traitement du Rachitisme, Paris, 8vo. 1797*) it was discovered that in this kind of syrup there was always a mercurial precipitate. The same has been discovered in the syrup of Bellet and Cuisiniere, although these syrups have been daily found successful in practice; indeed, when they are administered, the bottle ought to be shaken. After having mentioned to M. Bouillon La Grange all the ingredients which I wished to enter into the composition of antiscorbutic mercurial syrup with bitters, this eminent chemist published, in the *Journal du Pharmacien*, No. 180, a new method of preparing it. M. Salmade has repeated the formula in his *Traite sur les Maladies de la Lymphe;* and we think ourselves justified in subjoining it here also, leaving it to practitioners to simplify it still more, or to vary the proportion of the ingredients according to circumstances.

*Depurating Antiscorbutic Syrup.*

| | | |
|---|---|---|
| Gentian root, | —— | half an ounce. |
| Madder root, | —— | two drachms. |
| Quinquina, | —— | two drachms. |
| Horse radish, | —— | half an ounce. |
| Water cresses, | —— | q. s.* |
| Cochlearia, | —— | q. s. |
| Corrosive sublimate, | | two grains. |

The roots must be boiled with the quinquina in two pounds of water, until it is reduced to one; a pound and a half of sugar is to be added, and the whole clarified with the whites of two eggs. The mixture then assumes cise. My success was great, and numerous infants, whose spines or limbs were distorted, were restored to perfect symmetry of shape. Others, in whom the rachitis was confined to deformities in the bones of the cranium and chest, were completely cured.

A volume which I published on rachitis, was filled with these cures, which are to this day remembered in Paris, and particularly in the Faubourg St. Germain, where several families have been benefited by my treatment.

I might here add, that the method which I adopted for the treatment of rachitis was attended with every possible success when practiced by other physicians. The translations of my work into Italian and German, accompanied by various confirmatory facts by the translators, all concur in demonstrating the superiority of this treatment.

Other remedies have been described, both before and after the publication of my work; I can venture to assure the public, however, that neither the decoctions of aperitive plants, of madder, swallow-wort and hops, so highly extolled, nor extract of hemlock, by itself or combined with opium, nor the other bitter extracts, preparations of barytes and of lead, sea bathing, &c. can be put in competition with Bouvart's treatment, and which I adopted with some changes according to circumstances. If other remedies have sometimes been useful, it is because they were directed against simple albuminous or gelatinous swellings,* without any taint truly scrophulous; there are few practitioners who have not witnessed cases of this description.

Sometime ago Dr. Amelung published some observations upon the fortunate treatment of internal ulcerations, and upon those of the lungs in particular, constituting the last stage of pulmonary consumption, by sal Saturni and opium assumes the consistence of syrup.—On the other hand, the water cresses, the cochlearia, and the horse radish are bruised in a mortar; six ounces of juice are then obtained, to which eleven ounces of sugar are added, and the whole is warmed in a sand bath, in order to dissolve the sugar; this is mixed with the first syrup. The corrosive sublimate is dissolved in about a drachm of alcohol, and then intimately mixed with the syrup.

* In my *Memoire sur les Maladies de l'Epiploon*, printed in the volume of the Academy of Sciences for 1771, I have proved that there are swellings varying in the kind of substance of which they are composed, and that various remedies are consequently necessary for their cure. Chemistry having subsequently diffused some new light, with respect to animal humours, it is to be hoped that medical practitioners will profit from the circumstance.

um dissolved in a certain quantity of distilled fennel water. This remedy had been previously recommended by Dr. Hildebrand. But however respectable these authorities may be, as well as that of Dr. Hufeland, who published it in a Journal edited by himself, we are of opinion that, before believing such prodigies, this remedy ought to be subjected to additional trials by regular practitioners. Ulcerations of internal organs may result from causes of a diversified nature. How then can one and the same remedy cure the whole? This is beyond probability; but at present, nothing is in repute but new remedies, while several are allowed to sink into oblivion which have been approved by the most eminent of the profession, too often, because we do not know how to employ them so judiciously!

How useful would it be to found an Academy for preserving approved remedies from oblivion, and for determining the true cases in which they are applicable!

Hereditary pulmonary consumption being, in my opinion, of a scrophulous nature as well as rachitis, I shall never hesitate (having derived such great benefit in the treatment of the latter disease from mercurials combined with antiscorbutics and bitters) to apply them to hereditary phthisis, but under modifications relative to the more or less violent or advanced state of the disease and nature of the patient.

It has been already remarked, that milk diets are not proper for every kind of phthisis; and I have demonstrated, that it was chiefly in the constitutional scrophulous phthisis that we ought to prescribe aperients and depuratives of the above description, instead of milk diet, &c. The efficacy of this doctrine is now confirmed by numberless clinical observations. It must not be supposed, however, that the benefits alluded to were visible in patients far advanced in phthisis pulmonalis, unless the hereditary and scrophulous nature of the disease was announced by the external appearance of the body, and by original symptoms. Where is the disease that can be cured, when the organ wherein it is seated is in the last degree of destruction?

My success in the treatment of rachitis and of phthisis, as well as of other hereditary diseases, induced me to extend that treatment to two young patients subject to epileptic fits, which I considered as proceeding from a scrophulous cause, both of them having relations affected with the same disease, and not exempt from ricketty dispositions; positions; the treatment was long continued, suspended, or resumed, according to circumstances, and was attended with the happiest effects. One of these cases has been related, with all its minutiæ, in a work published several years ago by M. Salmade, M. D.* upon the diseases of the lymph. This author has also introduced a similar case, communicated to him by M. Brunet.

We find in the same work, the history of a child who had a large head, and the intellectual faculties almost annihilated, with scrophulous swellings of the lymphatic glands, who was cured by M. Salmade by means of the antiscorbutics combined with mercurials and bitters, as recommended by myself.† Now, after this successful treatment in the hereditary diseases which I have mentioned, can it be doubted that we may usefully extend the same method to cerebral affections, and to other diseases equally well known to be hereditary? Without doubt the treatment would be proportionably more efficacious if it were resorted to not only before the disease had made any great progress, but also when it began to manifest itself, as I have frequently experienced.

When I have been consulted by pregnant women, afflicted with any disease which might be transmitted to the child, or when her relations had a similar disease, I endeavoured to procure a good nurse for the child, and I prohibited the mother from nursing it herself, persuaded that the child must already have imbibed too much from her; and experience has shown, that nurses who were not too fat nor too strong, but who were slender and lively, and whose milk was a little transparent, were the best, particularly if they lived in the country in a good air; at least, I always found nurses of this description preferable to those who lived in cities, or who had been nurses in rich families.

I could mention several families in Paris, in which some children had died in dentition with unequivocal symptoms of rachitis, while others were preserved by having good nurses, some of whom, by my advice, used water-cresses, and some mercurial preparations, when the hereditary taint was too inveterate to yield to the efforts of Nature alone. But

---

* *Observations Pratiques sur les Maladies Rachitiques,* p. 168; a work full of clinical facts, equally curious and well authenticated.

† I have since collected various facts relative to diseases, in which the intellects were affected, and I have been confirmed in the utility of the same treatment.

But when there are slight defects from the birth, a good nurse will do them away entirely, at least greatly alleviate them. From this we may judge how essential it is to choose a good nurse, and how dangerous is the opinion hazarded by some writers, that mothers should always nurse their own children. This can only apply to such mothers as enjoy good health, and who have no disease which they can transmit to their offspring.

A good choice in marriages contributes not a little to attenuate hereditary taints, and without doubt the beneficial effects of this practice are frequently experienced in great cities, where the race is almost completely renewed by healthy men and women from the country. It is certain that we find true hereditary taints disappear by these means. This is the general opinion entertained in London; I have heard it said by several English physicians, and particularly by Pringle, that the Irish and Scotch revivify the nature of the inhabitants of London, who would otherwise become extinct.*

Persons living in certain districts, who have inherited endemic *goitres* from their parents, become free from them on removing to wholesome situations; but it is not till the third or fourth generation that the individuals are entirely freed from these appearances. In this way we may account for the disappearing of some hereditary complaints, and for the manner in which nature tends to rectify herself. Without this effect taking place, we could not assign the reason why whole families should not be extinct in a few generations.

Nature, however, is not always sufficient to cure her own deficiences, and often requires the assistance of medical science, for there are hereditary diseases which might produce not only the greatest deformities, but even the most disagreeable evils, if they were not prevented by judicious treatment. Now the first step to be taken with a child is to procure it good milk. In my work upon Pulmonary Phthisis, I have mentioned several curious and useful facts of this description. I have there given an account of an infant of high rank at Naples, who soon after birth appeared to be affected with complete rachitis. The head was enlarged in size, the spine was distorted, the clavicles were

---

* This remark may also apply to other cities, with respect to venereal diseases badly cured, and with respect to bad nursing and bad air. In these cases the men contract a scrophulous habit, and their children inherit the same disease.

were ill formed, and the belly was hard and very large. The parents of the child ascribed the cause of this disease to its nurse, and consulted various physicians of Paris and Montpellier. Messrs. Bouvart, Guenet, Borie, and myself were consulted at Paris; and Messrs. Chaptal, Lamure, Fouquet, and Farjon, at Montpelier. The first named physicians advised the nurse to use a mercurial syrup in small doses, but to be continued for a length of time, without any medicine for the child. Those of Montpelier recommended that both the nurse and child should be treated in this manner, with the addition of some small frictions of mercurial ointment.

For the sake of brevity, I shall not detail the nature or doses of the mercurial preparations which were prescribed. The nurse alone was treated as recommended by the Paris physicians, and the child was radically cured. Its limbs were completely developed, it grew tall and strong, and every symptom of rachitis disappeared.*

But when nurses are either unable or unwilling to submit to a medical regimen, or when the children have been several years ill, and there is a taint prevailing in their family, I have not hesitated to prescribe, as a preservative, the use of mild mercurials combined with antiscorbutics and bitters: a frequent use of the tepid bath, a regimen almost entirely vegetable, with no milk, and sometimes a cautery, and I never had reason to complain of the effects of this treatment. Motives of delicacy prevent me from publishing the names of the numerous families in which my practice has been successful.

I do not mean to state, however, that I always advise the same treatment in all hereditary diseases said to be scrophulous; when the syphilitic taint seems to be conspicuous, I strongly recommend mercurial remedies; I employ the antiscorbutics when the scrophulous taint is more apparent; and the bitters or even the feruginous remedies, with the cold bath, in weakly patients, where strength was chiefly required. When there was an excessive sensibility and irritability, I also combined mercurials with opium. I prescribed them internally to alleviate pain, in imitation of Cyrillo of Naples, who had derived much advantage from their external use in resolving external

* There are examples of venereal and scrophulous affections being cured by the milk of a goat, to which mercurial frictions had been applied upon a part of the skin previously stripped of the hair.

ternal scrophulous congeries. Lastly, I have employed the cautery according to the state of the patient; and sometimes I have continued the use of it until the age of puberty.

I have here given a detail of my observations upon the nature and treatment of hereditary scrophulous diseases. I have no doubt, that the advantages to be obtained by other practitioners, will be equal to those obtained by myself, and that they will also, in all probability, extend these advantages from their own experience.

